# A possible role of gas-phase electrophoretic mobility molecular analysis (nES GEMMA) in extracellular vesicle research

**DOI:** 10.1007/s00216-021-03692-y

**Published:** 2021-10-07

**Authors:** Stephanie Steinberger, Sobha Karuthedom George, Lucia Lauková, René Weiss, Carla Tripisciano, Ruth Birner-Gruenberger, Viktoria Weber, Günter Allmaier, Victor U. Weiss

**Affiliations:** 1 Institute of Chemical Technologies and Analytics, TU Wien, Getreidemarkt 9/164 CTA, A-1060 Vienna, Austria; 2 Center for Biomedical Technology, Department for Biomedical Research, Danube University Krems, Krems, Austria

**Keywords:** Gas-phase electrophoresis, Extracellular vesicle, Exosome, nES GEMMA, nES DMA, Mass spectrometry

## Abstract

The emerging role of extracellular vesicles (EVs) as biomarkers and their envisioned therapeutic use require advanced techniques for their detailed characterization. In this context, we investigated gas-phase electrophoresis on a nano electrospray gas-phase electrophoretic mobility molecular analyzer (nES GEMMA, aka nES differential mobility analyzer, nES DMA) as an alternative to standard analytical techniques. In gas-phase electrophoresis, single-charged, surface-dry, native, polydisperse, and aerosolized analytes, e.g., proteins or bio-nanoparticles, are separated according to their electrophoretic mobility diameter, i.e., globular size. Subsequently, monodisperse particles are counted after a nucleation step in a supersaturated atmosphere as they pass a focused laser beam. Hence, particle number concentrations are obtained in accordance with recommendations of the European Commission for nanoparticle characterization (2011/696/EU from October 18th, 2011). Smaller sample constituents (e.g., co-purified proteins) can be detected next to larger ones (e.g., vesicles). Focusing on platelet-derived EVs, we compared different vesicle isolation techniques. In all cases, nanoparticle tracking analysis (NTA) confirmed the presence of vesicles. However, nES GEMMA often revealed a significant co-purification of proteins from the sample matrix, precluding gas-phase electrophoresis of less-diluted samples containing higher vesicle concentrations. Therefore, mainly peaks in the protein size range were detected. Mass spectrometry revealed that these main contaminants belonged to the group of globulins and coagulation-related components. An additional size exclusion chromatography (SEC) step enabled the depletion of co-purified, proteinaceous matrix components, while a label-free quantitative proteomics approach revealed no significant differences in the detected EV core proteome. Hence, the future in-depth analysis of EVs via gas-phase electrophoresis appears feasible.

**Graphical abstract:**

**Figure:**
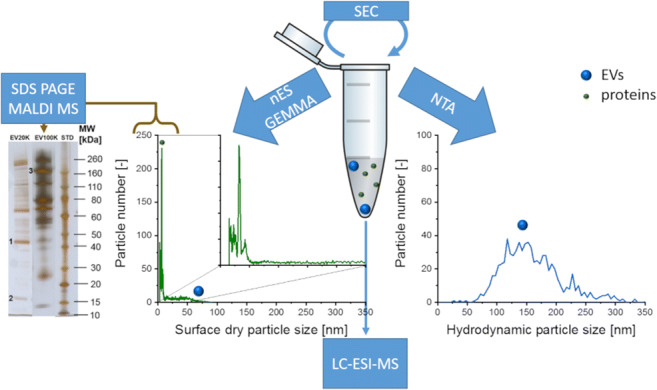
Platelet-derived extracellular vesicles (EVs)with/without additional size exclusion chromatographic (SEC) purification were subjected to nanoparticle tracking analysis (NTA) and gas-phase electrophoresis (nES GEMMA). The latter revealed presence of co-purified proteins, targetable via mass spectrometry (MS). MS also revealed that SEC did not influence EV protein content. To conclude, nES GEMMA is a valuable tool for quality control of EV-containing samples under native conditions allowing for detection of co-purified proteins from complex matrices.

**Supplementary Information:**

The online version contains supplementary material available at 10.1007/s00216-021-03692-y.

## Introduction

Extracellular vesicles (EVs) consisting of a lipid bilayer encapsulating an aqueous lumen are bio-nanoparticles secreted by cells. They have been described in various biological fluids, such as urine, blood, and saliva, and are typically defined by a size range of 30–1000 nm [1]. Originally, EVs were suggested to transport waste material out of cells [2]. Meanwhile, however, there is ample evidence that EVs are essential in many physiological processes, including intercellular communication and cellular homeostasis, next to their transport function for proteins, lipids, DNA, and RNA [3, 4]. Furthermore, they play essential roles in immunomodulation, coagulation, and thrombosis, as well as tumor metastasis [5–7]. Depending on the physiological state of their parent cells, their tissue of origin, and their microenvironment, differences in the composition and function of EVs occur [3, 8–10].

Due to their availability in body fluids and their composition that reflects the state of their parent cells, EVs have been suggested as valuable biomarkers [11–14]. However, for this application, the enrichment of EVs out of complex biological matrices and the optimization of corresponding preparation protocols are required. Various analytical strategies have been described for the isolation of EVs from biological fluids or cells to date, including (ultra)centrifugation [15, 16], chromatographic approaches with a special focus on affinity and size exclusion chromatography (SEC) [17], (ultra)filtration [18], and precipitation [19]. Commercially available EV isolation kits, which combine centrifugation, precipitation, and enzymatic digestion of samples, are widely applied due to their claimed straightforward usage, speed of analysis, and availability [20–22]. However, all these methods share similar limitations regarding EV recovery and purity due to only partial EV enrichment, co-purification of non-vesicular components, and analyte loss based on EV interactions with reagents or supporting materials [23]. The lack of a universal marker for all EV populations often requires multiple purification rounds applying orthogonal methods in order to obtain EV preparations of high purity, which is associated with a loss of EV material.

Besides challenges in EV enrichment, the characterization of these bio-nanoparticles is a challenging task, because vesicle numbers are often low, and native particle sizes are in a range that is not easily targetable. To date, prevalent characterization techniques include flow cytometry and nanoparticle tracking analysis (NTA), often utilized in combination with laser-induced fluorescence, laser light scattering, microscopic techniques, and affinity-based methods [24, 25]. It is of note that some of the aforementioned methods yield information on bio-nanoparticle size and size distribution (e.g., microscopy techniques), however, at the cost of a large number of necessary analyses in order to obtain a sufficiently large, statistically valid dataset. Other methods do not detect smaller sized sample components besides vesicles due to inherent constraints, e.g., characterization techniques based on light scattering. Despite these shortcomings, EV material prepared and characterized in such ways has been applied for comprehensive studies targeting vesicle protein, genome, and lipid content [5, 10, 26].

In this manuscript, we focus on the applicability of nano electrospray gas-phase electrophoretic mobility molecular analysis (nES GEMMA) as a possible supplement to established analytical techniques for EV characterization. In addition to the acquisition of size distributions of surface-dry analytes and particle counts as suggested by the European Commission for characterization of material in the nanometer size range (2011/696/EU from October 18th, 2011), nES GEMMA offers the possibility of collecting and concentrating EVs on supporting materials. Hence, analyses with orthogonal methods, such as mass spectrometry [27], atomic force microscopy [28], dot blot analysis [29, 30], or cell culture [31] investigations, are feasible. Additionally, analyte molecular weight values can be estimated from nES GEMMA data [32].

nES GEMMA is based on the separation of single-charged nano-sized analytes in the gas phase according to their electrophoretic mobility diameter (EM diameter) and was first described by Kaufman and colleagues in 1996 [33]. Single-charged nanoparticles are obtained after electrospraying analytes from a volatile electrolyte solution (nES process) with subsequent drying of the EV-containing droplets and charge reduction in a bipolar atmosphere induced by a ^210^Po α-particle source or similar [34–36]. Subsequently, the separation itself occurs inside a nano differential mobility analyzer (nDMA) within a high laminar flow of particle-free, compressed air and an orthogonal, tunable electric field. Variation of the field strength enables particle separation—monomobile analyte fractions are obtained after passage of the nDMA. Subsequently, particles act as nuclei for condensation in a supersaturated atmosphere of n-butanol or water in the ultrafine condensation particle counter (CPC) of the instrument. Particles are counted as they induce laser light scattering. Such a setup is also known under several other names—nES Differential Mobility Analyzer (nES DMA) [37, 38], LiquiScan ES [39], Macro Ion Mobility Spectrometer (MacroIMS) [40], or Scanning Mobility Particle Sizer (SMPS) [41]. However, for matters of consistency with work from our group as well as others, we have chosen to remain with the term nES GEMMA.

Previous studies have demonstrated the applicability of nES GEMMA for the characterization of liposomes—nanovesicles consisting of a lipid bilayer with an aqueous core—in terms of vesicle size and heterogeneity of preparations [42]. In addition, a first study already reported nES GEMMA also for EVs [43]. However, in this study, only particles exceeding 20 nm EM diameter (> 1.5 MDa on the protein scale; [32]) were analyzed with adjusted instrument settings. Furthermore, nES GEMMA has already been successfully applied for lipoprotein particle characterization [42, 44, 45].

nES GEMMA enables to analyze a size range from single digit up to several hundred nanometers, and instrument settings have to be adapted for smaller and larger sample components (refer to Supplementary Information Figure S1 for a schematic overview on the targetable analyte size range via nES GEMMA and NTA, respectively). In order to analyze smaller sized analytes such as small proteins, a high sheath flow inside the nDMA yields highly resolved peaks. At the same time, however, the targetable size range of bio-nanoparticles is significantly reduced. A large size range, on the other hand, comes at the cost of poor peak resolution for homogeneous sample components, such as proteins. In the case of heterogeneous analytes such as intact EVs, no additional peak broadening occurs for low sheath flow values.

Focusing on EV-containing samples purified from complex matrices, this results in two possible analysis approaches especially when taking concentration differences between smaller and larger sample components into consideration. nES GEMMA can either focus on analytes up to a few 10 nm EM diameter (proteins, genomic material, etc.) with good resolution, or on EVs, excluding the lower size range of the spectrum for analysis ([43]). Concentrating on the first approach, it was the aim of our study to demonstrate that nES GEMMA yields valuable information concerning the purity of extracellular vesicle-containing samples, especially in the region below 20 nm EM diameter. Particles in this size/molecular weight range are possibly overlooked when relying on other analysis methods like NTA alone usually enabling detection of particles larger than several 10 nm hydrodynamic diameter. Especially for proteomics and immunoanalytical approaches targeting EV-associated proteins, sample purity and protein co-purification are crucial, and we suggest that nES GEMMA can contribute significantly to the quality control of EV-containing samples.

## Materials and methods

### Isolation of extracellular vesicles

Medical grade platelet concentrates from healthy donors were applied for the isolation of EVs. Concentrates were obtained from the Clinic for Blood Group Serology and Transfusion Medicine (Medical University Vienna, Vienna, Austria) as approved by the Ethics committee of Danube University Krems (Krems, Austria) (ethics votum number ECS2177/2015). Written informed consent was obtained from all donors in our study. EVs were isolated either using the “Total Exosome Isolation Kit from plasma” (Invitrogen, Carlsbad, CA, USA) based on a low-speed centrifugation step after EV precipitation or a two-step ultracentrifugation approach at 2 × 10^4^*g* and 1 × 10^5^*g*, respectively [46]. Prior to ultracentrifugation, platelets were removed from samples at 2.5 × 10^3^*g*. Following ultracentrifugation, the pellets were re-suspended in phosphate-buffered saline (PBS; Life Technologies, Paisley, UK) [46]. PBS was filtered (0.1 μm Minisart syringe filter, Sartorius Stedim Biotech, Goettingen, Germany) prior to use.

In an additional approach, EVs after ultracentrifugation (500 μL volume) were further processed using size exclusion chromatography (qEV, Izon Science, Burnside, Christchurch, New Zealand). Fractions (500 μL each) were collected and fractions containing Annexin V-positive (AnxV^+^) EVs according to flow cytometry were pooled and centrifuged at 2 × 10^4^*g* and 1 × 10^5^*g*, respectively, resulting in an EV20k SEC and an EV100k SEC sample.

### Flow Cytometric characterization of platelet-derived EVs

EV suspensions were diluted in PBS to a protein concentration of 1 μg mL^−1^. Aliquots of 100 μL were stained for 15 min at RT in the dark with fluorescein isothiocyanate-conjugated Annexin V (Becton Dickinson, Eysins, Switzerland) as marker of phosphatidylserine, as well as with phycoerythrin cyanin 7 (PE-PC7)-conjugated anti-CD41 antibody (Beckman Coulter, Brea, CA, USA) as platelet marker. All antibody conjugates were centrifuged at 1.7 × 10^4^*g* for 10 min at ambient temperature prior to use to remove aggregates. Stained samples were further diluted fivefold in PBS and analyzed on a CytoFLEX LX flow cytometer (Beckman Coulter). Fluorescent-green silica particles (1 μm, 0.5 μm, 0.1 μm; excitation/emission 485/510 nm; Kisker Biotech, Steinfurt, Germany) were used for calibration. The triggering signal was set to violet side scatter and the EV gate was set as shown previously [46–48]. Data were analyzed using the Kaluza Software (Beckman Coulter).

### nES GEMMA measurements

Measurements on a nES GEMMA require analytes dissolved in a volatile electrolyte solution. Therefore, PBS of EV preparations was exchanged to 40 mM ammonium acetate, pH 8.4 (Sigma-Aldrich, St. Louis, MO, USA) with 10k MWCO filters (Pall Laboratory, Port Washington, NY, USA) at 9.3 × 10^3^*g*. Subsequently, the EV-containing samples were further diluted 1:10 (v:v) by means of an ammonium acetate (40 mM, pH 8.4) solution. The nES occurred at constant pressure of 4 psid (0.28 bar) and in average 2.0 kV spray voltage at the tip of a 25-μm-inner diameter, cone-tipped capillary [49] in the nES aerosol generator (model 3480, TSI Inc, Shoreview, MN, USA). Subsequently, a gas flow of 1.0 L min^−1^ dried air and 0.1 L min^−1^ of carbon dioxide was used to transport the polydisperse aerosol through the bipolar atmosphere inside the charge reduction chamber induced by a ^210^Po α-particle source. Concomitantly, the electrolyte solution evaporated and single-charged, surface-dry analytes were separated in a nano differential mobility analyzer (nDMA, model 3080, TSI Inc) applying a sheath flow of 8.0 L/min. Following separation, nanoparticles were counted by laser light scattering after having induced nucleation in a n-butanol saturated atmosphere in an ultrafine condensation particle counter (model 3776, TSI Inc). The measuring range encompassed particles from 3.0 to 91.4 nm EM diameter. Five scans (adjustment of the nDMA separation voltage—190 s scan time, 20 s for voltage re-setting) were combined via their median to obtain a corresponding nES GEMMA spectrum.

### Nanoparticle tracking analysis (NTA)

For NTA, the samples were diluted 1:10,000 (v:v) and 1:50,000 (v:v), for SEC samples 1:1000 (v:v) in high-purity water (18.2 MΩcm resistivity at 25 °C, MilliQ-System, Merck, Darmstadt, Germany), or 40 mM ammonium acetate solution, pH 8.4 and immediately measured after dilution. The measurements were performed with a Zetaview PMX120 (ParticleMetrix, Meerbusch, Germany) at 22 °C. Each measurement consists of 11 scattering measurement positions. The minimum brightness of the expected particles was set to auto, the particle size range was set to 5–200 nm, the shutter was set to 100, the minimum tracelength was set to 15, and the frame rate was set to 15 (arbitrary units, each). On average, the concentration of EV samples was 10^11^–10^12^ particles per milliliter, while SEC samples only contained around 10^8^–10^9^ particles per milliliter. The obtained data was analyzed with the corresponding instrument software (ZetaView 8.05.05 SP2) to calculate mean, standard deviation, and size distribution.

### MALDI MS

One approach to identify proteins in EV20k and EV100k samples was based on SDS-PAGE, in-gel tryptic digest, and MALDI MS. A NuPAGE (Invitrogen, Waltham, MA, USA) 12% Bis-Tris gel electrophoresis with a sample concentration of 0.5 μg total protein content per lane was performed, using pre-stained molecular weight markers (NovexSharp pre-stained, Invitrogen, Waltham, MA, USA) followed by a MS-compatible silver staining based on [50]. Visible protein bands were excised and subjected to an overnight tryptic in-gel digest [51]. For a better digestion efficiency, a trypsin/lysC mixture with the concentration of 0.1 ng/μL from Promega (Madison, WI, USA) was used. In addition, the digested peptides were purified with a ZipTip_C18_(Merck) purification protocol. The purified peptides were directly eluted with a 3 mg mL^−1^α-cyano-4-hydroxycinammic acid (CHCA) matrix (acetonitrile/water/TFA (60:40:0.1) (v:v:v)) solution on a MTP 384 stainless steel MALDI MS target. All measurements were performed on an UltrafleXtreme mass spectrometer (Bruker Daltonics, Bremen, Germany) in positive RTOF (reflector time-of-flight) and tandem TOF/RTOF mode. For the acquisition of the mass spectra in the RTOF and tandem TOF/RTOF mode, an accumulation of 6000 and 10,000 laser shots, respectively, at a laser power of 39% and 1 kHz in a range of 500–3500*m*/*z* was performed. The obtained data was analyzed with the manufacturer’s software flexAnalysis (v.3.4 Build 57) and the protein identification was implemented with the Mascot Search Engine (MatrixScience, London, UK). As a database, the NCBI protein database for *Homo sapiens* was chosen, as well as carbamidomethylation on Cys as a fixed modification. Acetylation on the protein N-terminus, oxidation on Met, phosphorylation on Ser and Thr, and deamidation on Asn and Gln were set as variable modifications. Furthermore, the missed cleavages were set to 2, the molecular mass tolerance to ±0.3 Da, and the fragment ion tolerance to ±0.5 Da.

### LC-ESI-MS

Three independent preparations of EV samples before and after SEC corresponding to 600-ng protein content were subjected to clean up via SDS-PAGE, reduction, alkylation, and in-gel tryptic digest. One-third of the digests was analyzed by LC-MS/MS. Chromatography was carried out on an Ultimate 3000 RCS Nano Dionex system (Thermo Scientific, Waltham, MA, USA) equipped with an Ionopticks Aurora Series UHPLC C18 column (250 mm × 75 μm, 1.6 μm) (Ionopticks, Parkville, Australia). Solvent A was 0.1% formic acid in water and solvent B acetonitrile containing 0.1% formic acid. A total LC-MS/MS run per sample lasted for 136.5 min with the following gradient: 0–5.5 min: 2% B; 5.5–65.5 min: 2–17% B; 65.5–95.5 min: 25–37% B; 105.5–115.5 min: 37–95% B; 115.5–125.5 min: 95% B; 125.5–126.5 min: 95–2% B; 126.5–136.5 min: 2% B at a flow rate of 400 nl min^−1^ and a column temperature of 50 °C. The timsTOF mass spectrometer (Bruker Daltonics) was operated in positive ion mode with enabled trapped ion mobility spectrometry (TIMS) at 100% duty cycle (100-ms cycle time). Scan mode was set to parallel accumulation-serial fragmentation (PASEF) for the scan range of 100–1700*m*/*z*. Source capillary voltage was set to 1500 V and dry gas flow to 3 L min^−1^ at 180 °C. LC-MS/MS data was subjected to MaxQuant (v1.6.17.0) [52–54] Andromeda search of the Uniprot human database containing common contaminants (20,467 entries) using 1% PSM (peptide spectrum matches) and protein FDR (false discovery rate) as threshold for identification (including carbamidomethylation on Cys as fixed, oxidation on Met, and acetylation on protein N-terminus as variable modifications, minimum peptide length 7 amino acids) and minimum 2 ratios of unique and razor peptides for label-free quantification (LFQ).

LFQ values were log2 transformed and subjected to statistical analysis using Perseus (v1.6.12.0.) [55]. Prior to statistical testing, the matrix was filtered for common contaminants and to keep only those proteins with reported valid values in at least 3 samples in at least one group. Missing values were then imputed from a normal distribution with a width of 0.3 and a downshift of 1.8. Histograms of intensities of quantified proteins are shown in Supplementary Information Figure S2 suggesting a normal distribution, where measured proteins are depicted in blue while imputed proteins are depicted in red. A two-sample*t*-test corrected for multi-testing was performed between the groups (permutation-based FDR 5%, S0 = 2). The MS proteomics datasets were deposited to the ProteomeXchange Consortium via the PRIDE partner repository [56] with the dataset identifier PXD024760 (Reviewer account details: Username: reviewer_pxd024760@ebi.ac.uk; Password: pGPaJoKy).

## Results and discussion

**Fig. 1:**
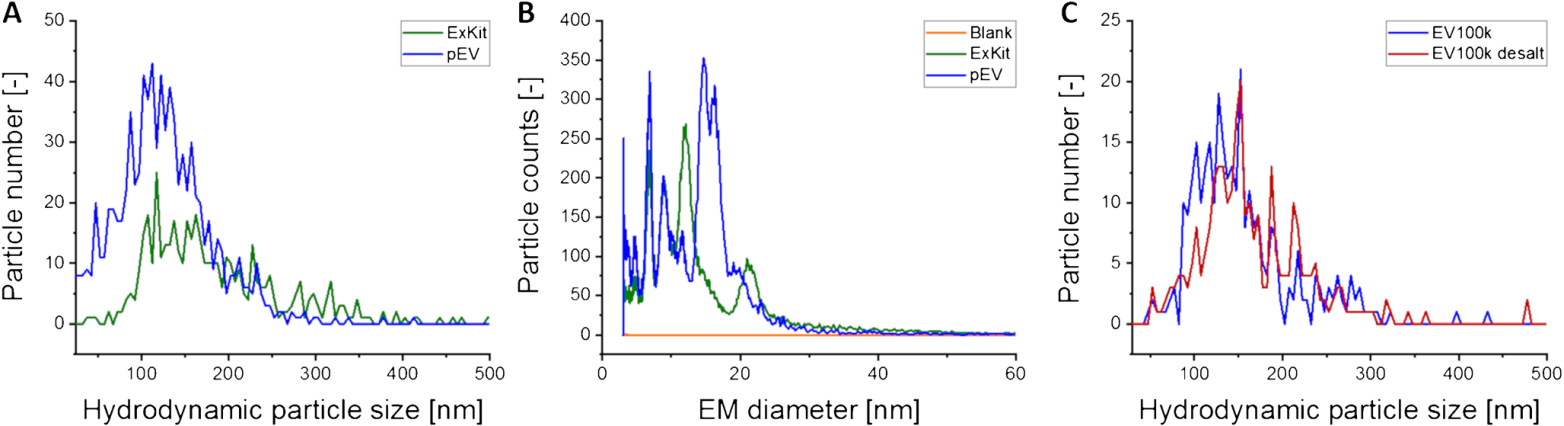
Comparison of two EV isolation techniques and investigation of the impact of solvent exchange on particle numbers. Comparison of the hydrodynamic particle size distribution (NTA-derived, **A**) and the dry particle size distribution (nES GEMMA-derived, **B**) of EVs obtained applying two isolation techniques—ultracentrifugation(pEV) and an exosome isolation kit (ExKit). The effect of electrolyte exchange via a 10-kDa MWCO filter (**C**) on the hydrodynamic size distribution and particle number of the samples, as evaluated by NTA

Comprehensive EV research relies on well-characterized, highly purified vesicle material. Focusing on two preparation and enrichment techniques—ultracentrifugation and application of an EV precipitation kit—we started our work dealing with EVs purified from human medical platelet concentrate. Enriched EV fractions were characterized using NTA, which detected particles of approximately 120 nm (centrifugation) and 160 nm (precipitation) hydrodynamic diameter (Fig. 1A). However, subjecting the corresponding samples to gas-phase electrophoresis revealed the presence of material in the EM diameter range, which was associated with free proteins at very high concentrations. In contrast, signals for larger assemblies (EVs) were very low (Fig. 1B) and the occurrence of free proteins in such high concentrations precluded nES GEMMA of less-diluted samples which would putatively have been led to increased particle counts in the higher EM diameter range.

Focusing on the origin of proteins detected via nES GEMMA, we reasoned that these either might be co-purified with EVs from solution or might originally be EV-associated (attached to the EV surface, encapsulated in the vesicle aqueous core, or were a part of the lipid EV bilayer). In the latter case, detection of free proteins in such high concentrations especially in combination with low signals obtained in the higher EM diameter range probably indicates loss of EV integrity during sample preparation.

In order to analyze samples via nES GEMMA, the removal of non-volatile sample buffer components is a prerequisite. In this context, interaction of vesicles with membrane material applied for electrolyte exchange, the forces exerted on EVs during this process, or electrolyte-dependent changes in osmolarity or pH might have led to vesicle damage. Thus, EVs would have been no longer detectable due to their removal from samples. As a consequence, proteins recorded in nES GEMMA spectra would have been released from vesicles upon rupture. On the other hand, sample preparation and nES GEMMAs of intact liposomes as well as other non-covalently bound macromolecule assemblies, such as glycoproteins and lectins, have already been published [30, 42, 45, 57–59]. Therefore, analyte transition from the liquid to the gas phase in nES GEMMA is known to occur under native conditions. Hence, the idea that protein signals detected with nES GEMMA originated from material released from EVs upon vesicle rupture during storage and electrolyte exchange appeared unlikely. Nevertheless, this possibility was further investigated.

Taking corresponding samples to our NTA setup revealed that EVs were present after the necessary exchange of electrolyte solutions, although reduced in overall particle numbers by about 90% as deduced from NTA measurements. Particle hydrodynamic diameter values remained comparable prior to and after exchange of electrolyte solutions possibly excluding preferential loss of vesicle subclasses (Fig. 1C; based on different sample dilutions in order to obtain comparable analyte numbers).

Based on this observation, the idea of co-purification of proteins during the enrichment of EVs gained significance. Such a co-purification would usually go unnoticed, as most analysis methods other than nES GEMMA concentrate on a size region of EVs rather than focusing on co-purified proteins (refer to Supplementary Information Figure S1 for a schematic comparison of the nES GEMMA and NTA sizing range). It was thus our intention to demonstrate such a co-purification via additional purification steps, after a detailed analysis of co-purified sample components.

### Identification of main protein components of EV preparations

**Fig. 2:**
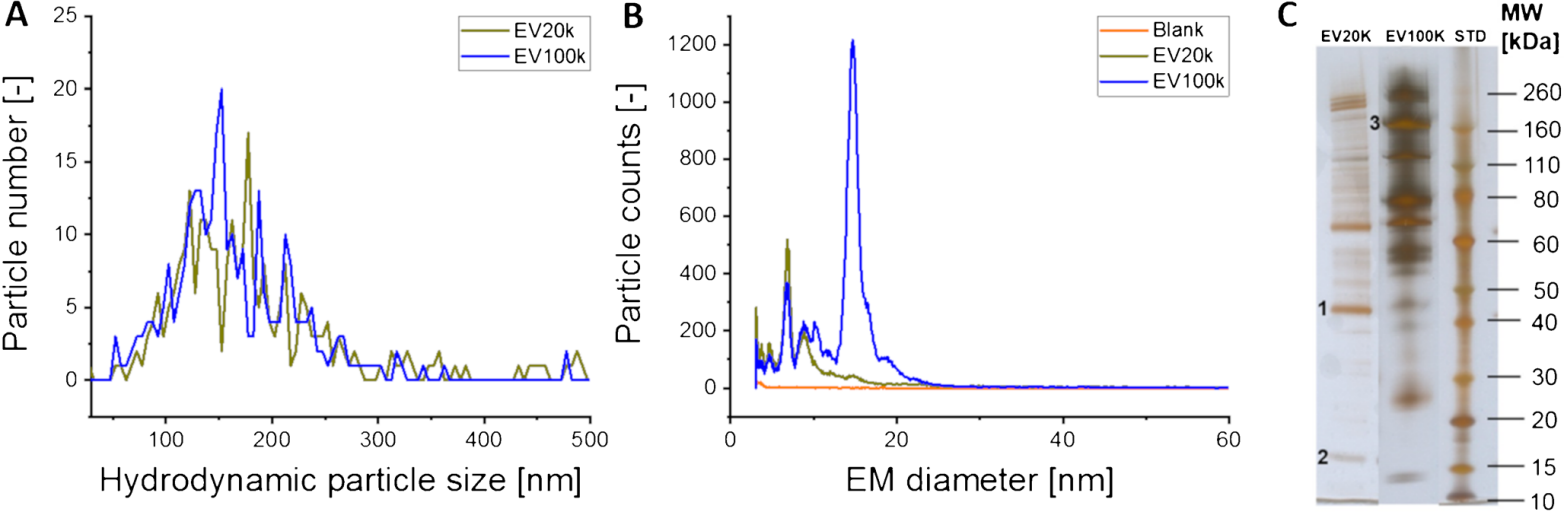
Characterization of larger (EV20k) and smaller (EV100k) EVs obtained via centrifugal isolation. Hydrodynamic particle size distribution (NTA-derived, **A**) and dry particle size distribution (nES GEMMA-derived, **B**) of the EV-containing fractions were measured. SDS-PAGE (**C**) for further analysis with MALDI MS/MS revealed differences in the protein composition of the fractions in comparison to a protein standard (STD)

During EV enrichment via ultracentrifugation, at 2 × 10^4^*g*, larger vesicle components (EV20k, “microvesicles”) are pelleted, whereas at 1 × 10^5^*g*, smaller sized material (EV100k, “exosomes”) is obtained. Following these enrichment steps, samples were characterized using NTA and nES GEMMA (Fig. 2A and B), as presented in the previous section. Additionally, proteinaceous sample compounds were analyzed using SDS-PAGE (Fig. 2C), a subsequent tryptic in-gel digestion, and MALDI MS/MS identification. As expected, SDS-PAGE disclosed a multitude of proteins contained in EV samples. Lanes were excised and an in-gel digestion was carried out overnight prior to Zip Tip purification and mass spectrometric analysis by means of a MALDI-TOF/RTOF-MS instrument. Such an approach identified three protein components with 95% confidence—β-actin (like) protein (Supplementary Information Figure S3A), haemoglobin (Supplementary Information Figure S3B), and α-2-macroglobulin(Supplementary Information Figure S3C). These proteins are already described in ExoCarta (http://www.exocarta.org, date of retrieval: 15.02.2021) as being associated with platelet-derived EVs. However, these proteins are also blood compounds involved in immune response, coagulation, and platelet function [26, 60, 61]. Thus, whether these proteins were originally EV-associated (encapsulated, part of the lipid bilayer or membrane attached) or were just co-purified still remained elusive via our chosen analytical approach.

**Fig. 3:**
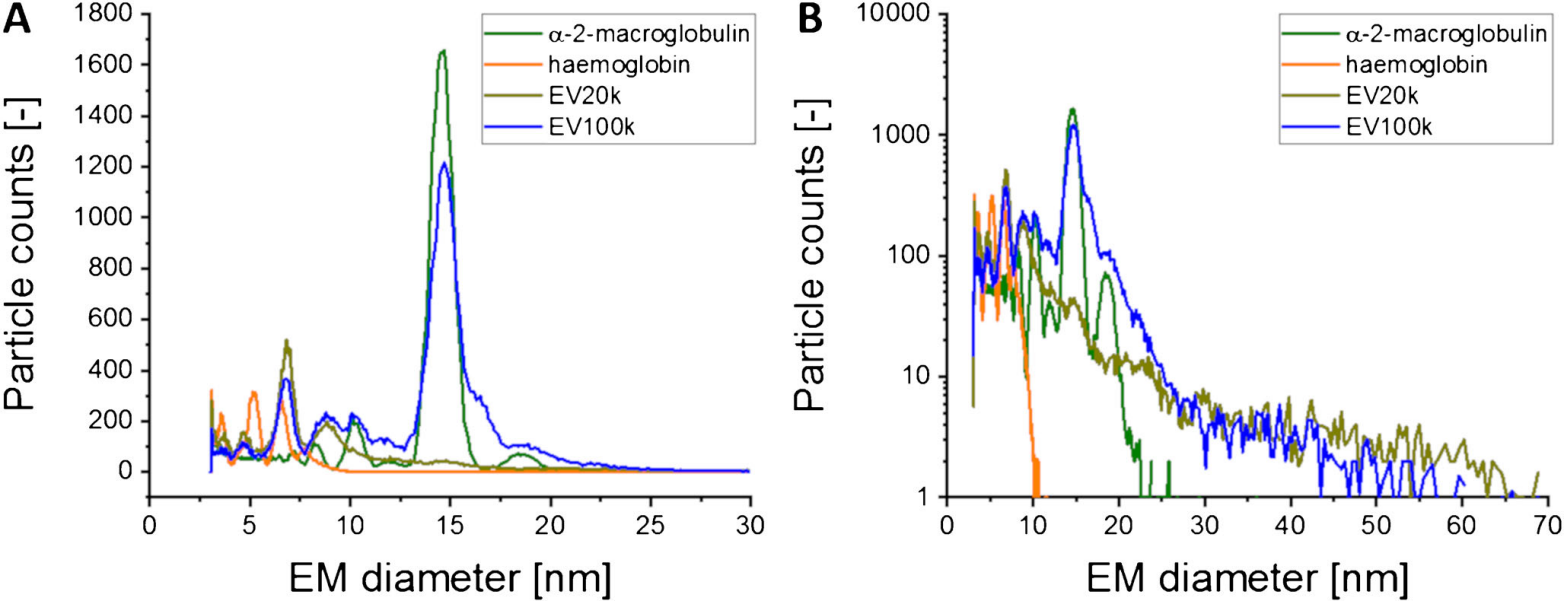
Relation of nES GEMMA spectra of EVs and identified contaminants. Two contaminants, α-2-macroglobulin and haemoglobin, common in human blood and identified via SDS-PAGE, in-gel digest, and MALDI MS/MS, were compared to EV isolates. Data is shown in linear scale (**A**) and logarithmic scale (**B**) to emphasize nES GEMMA signals obtained for vesicles in the higher EM diameter range

Next, to demonstrate that haemoglobin and α-2-macroglobulin were part of nES GEMMA spectra, we analyzed these proteins both individually and in a mixture via gas-phase electrophoresis. Originally according to Bacher et al. [32], the molecular weight of proteins correlates with their EM diameter, resulting in a theoretical EM diameter for haemoglobin (hetero-dimer, approx. 64 kDa) and α-2-macroglobulin (homo-tetramer, approx. 720 kDa) of 6.7 nm and 14.9 nm, respectively. Measurements of both proteins with nES GEMMA led to experimentally determined EM diameters of 6.6 ± 0.3 nm and 14.6 ± 0.2 nm (*n* = 3 measurements), respectively, which corresponds well with peaks in the pattern of the EV-containing samples (Fig. 3).

### Depletion of proteinaceous sample components and its effect on a proteomics profiling approach

**Fig. 4:**
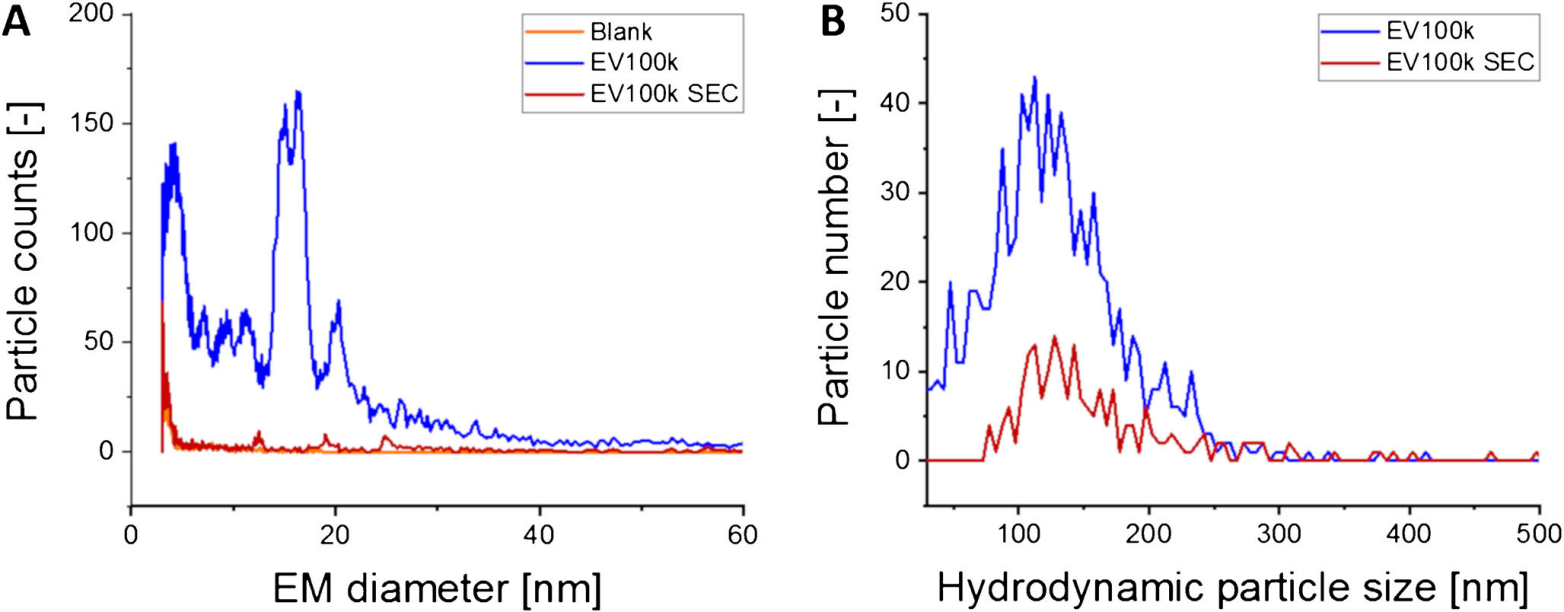
Influence of SEC on EV preparations obtained after ultracentrifugation. The dry particle size distribution (nES GEMMA-derived, **A**) and the hydrodynamic particle size distribution (NTA-derived, **B**) of smaller EVs (EV100k) were measured and reveal loss of contaminating material in the low EM diameter range as well as a reduction of detected vesicles

As the high concentration of proteinaceous material in EV samples caused considerable problems in comprehensive vesicle analysis, SEC was introduced as an additional purification step after ultracentrifugation. Focusing on EV100k preparations, a comparison between ultracentrifugation alone and a combination of ultracentrifugation and SEC clearly indicated a depletion of low EM diameter material in nES GEMMA (Fig. 4A) following SEC. At the same time, NTA confirmed the presence of EVs after the additional SEC step, although a loss of vesicles by about 60% (particle number based) occurred, which was possibly due to vesicle interaction with the chromatographic material and/or shear forces within the separation column (Fig. 4B). This finding renders ruptured vesicles as the origin of detected proteins highly improbable, as in this case also protein peaks should have been detected in the last nES GEMMA sample of the described workflow.

**Fig. 5:**
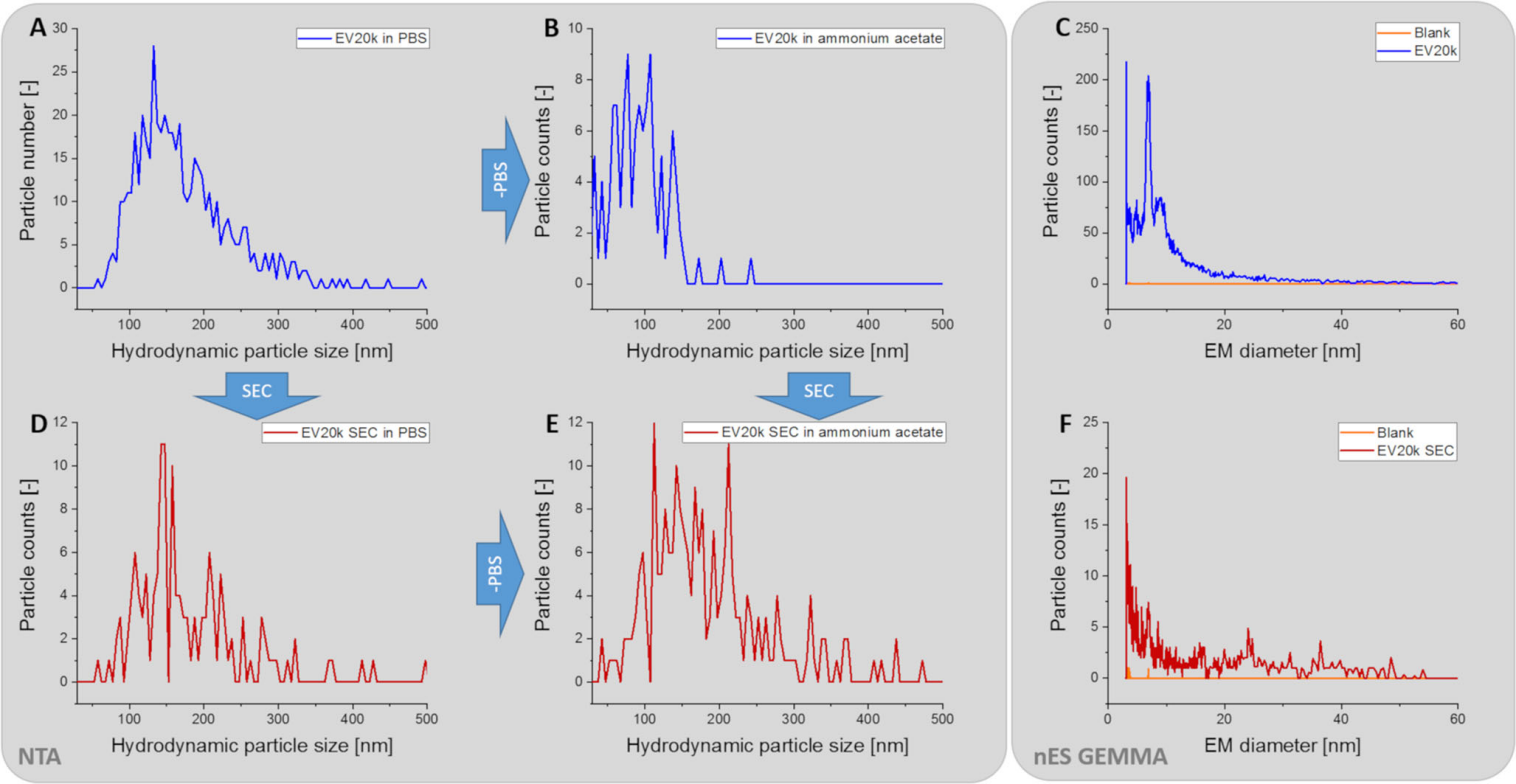
Influence of solvent exchange via filtration on EV samples. The hydrodynamic size distribution was measured before and after the exchange of PBS to an ammonium acetate solution (**A** and **B**; **D** and **E**) for the larger EVs before and after SEC. The corresponding nES GEMMA measurements (**C**, **F**) display the dry particle size distribution

An additional complete dataset demonstrating our hypothesis of co-purifed proteins detected in the low EM diameter range upon nES GEMMA is presented in Fig. 5. Presence of EVs in samples is confirmed via NTA measurements. Exchange of the electrolyte solution to ammonium acetate resulted in a reduction of vesicle counts by ≥60%, as demonstrated by NTA. At the same time, protein-related peaks were detectable by means of nES GEMMA. Processing the original sample via SEC resulted likewise in a loss of vesicles by about 60%. However, EV occurrence could still be confirmed by NTA measurements. Another step of electrolyte exchange of this SEC purified sample also led to vesicle loss as detected by NTA measurements. However, at the same time, nES GEMMA failed to detect protein-related peaks.

**Fig. 6:**
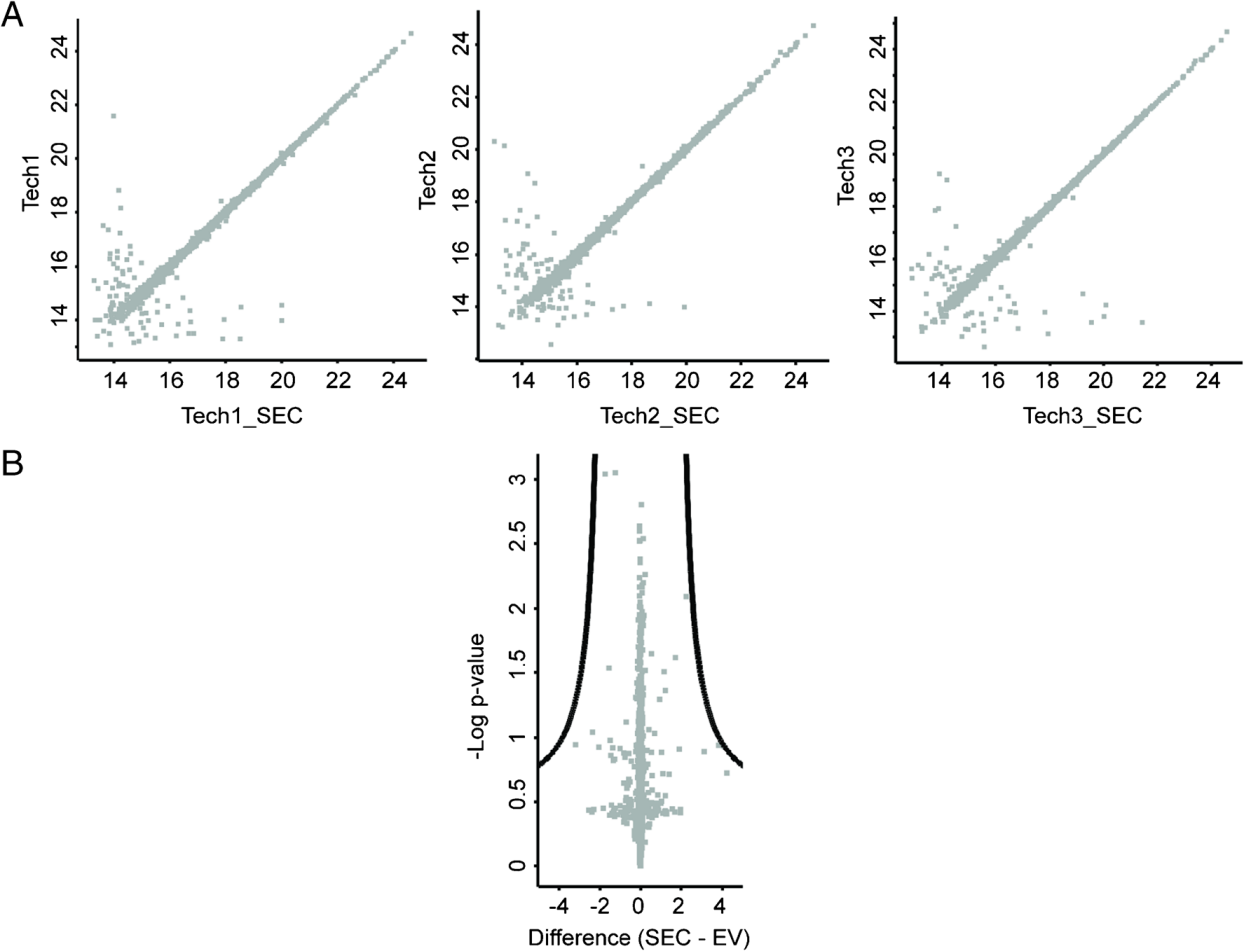
LC-ESI-MS proteomics analysis of smaller EVs (100 k) before and after SEC isolation. **A** Scatter plot of three independent EV preparations (named Tech 1, Tech 2, Tech 3) before and after SEC shows that log2 LFQ intensities of common proteins depict a highly linear correlation (**A**). **B** Volcano plot of EV samples before (EV) and after SEC (SEC) depicts −log *p* values after multi-testing control (FDR 5%) of a two-sided*t*-test (S0 = 2) versus the differences between mean log2 LFQ intensities before and after SEC, respectively. Samples show a high similarity with no significant difference in the proteome of the EVs due to the application of an additional SEC isolation step (**B**)

We next analyzed whether additional sample pretreatment steps such as SEC would significantly influence the protein content of EVs. Focusing on an EV preparation not showing any co-purified proteins in the low EM diameter range via nES GEMMA, we compared LC-MS/MS data of a sample with and without additional SEC, employing three technical replicates of the preparations (samples Tech 1–Tech 3). We identified and quantified between 2182 and 2217 proteins in each sample when injecting similar peptide amounts. Overall, 1968 proteins were common in all 6 samples; 123 proteins in 5, 30 proteins in 4, and 8 proteins in only 3 samples after filtering for at least three valid values in at least one group. As demonstrated in Fig. 6, no significant difference in detected proteins (overall *n* = 2129) could be obtained before and after SEC. The complete list of proteins can be found in Supplementary Information Table S1. Moreover, about 2000 common proteins in the individual preparations (sample Tech 1: 2054; sample Tech 2: 2057; sample Tech 3: 2069) before and after SEC followed a perfect linear correlation.

Hence, we reasoned that application of SEC is a suitable method for reduction of co-purified proteinaceous sample components while vesicle-associated proteins are not affected by SEC, despite a significant loss of overall vesicle numbers.

## Conclusion

The heterogeneity of EVs makes their isolation, purification, and characterization a challenging task. Characterization of EVs with nES GEMMA is an alternative option in addition to current characterization techniques, such as flow cytometry, NTA, laser light scattering, microscopic techniques, and affinity-based methods [24, 25]. Applying nES GEMMA, we observed co-purification of proteins in high quantities, adding additional complexity to sample characterization. The main co-enriched components were identified via a SDS-PAGE, in-gel digest, and MALDI MS/MS approach. nES GEMMA measurements corroborated the MS-based protein identification.

Previous studies have questioned the influence of SEC on vesicle membrane integrity and surface protein patterns [26, 55]. Using nES GEMMA, we found that an additional SEC step following EV isolation by ultracentrifugation depleted the putatively co-isolated proteins. At the same time, SEC appears to have no influence on the EV proteome as such, even though it leads to a loss of approximately 60% of EVs as determined via NTA.

To conclude, nES GEMMA is a valuable approach for quality control of EV-containing samples under native conditions, as it allows for the detection of co-purified proteins from complex matrices.

## Supplementary information

ESM 1 (DOCX 802 kb)

ESM 2 (XLSX 444 kb)
