# Supplementary material for: A possible role of gas-phase electrophoretic mobility molecular analysis (nES GEMMA) in extracellular vesicle research
Source: Anal Bioanal Chem. 2021 Oct 7;413(30):7341–52. doi: 10.1007/s00216-021-03692-y (PMC8626398; doi:10.1007/s00216-021-03692-y)
Supplement: Supplementary file 1 — (DOCX 802 kb) [file 216_2021_3692_MOESM1_ESM.docx]

ELECTRONIC SUPPLEMENTARY INFORMATION

**A possible role of gas-phase electrophoretic mobility molecular analysis (nES GEMMA) in extracellular vesicle research**

Stephanie Steinberger^1^, Sobha Karuthedom George^2^, Lucia Lauková^2^, René Weiss^2^, Carla Tripisciano^2^, Ruth Birner-Gruenberger^1^, Viktoria Weber^2^, Günter Allmaier^1^, Victor U. Weiss^1^

*^1^ Institute of Chemical Technologies and Analytics, TU Wien, Vienna, Austria*

*^2^ Center for Biomedical Technology, Department for Biomedical Research, Danube University Krems, Krems, Austria*

**Corresponding author:** Victor U. Weiss, Institute of Chemical Technologies and Analytics, TU Wien (Vienna University of Technology), Getreidemarkt 9/164 CTA, A-1060 Vienna, Austria

e-mail: victor.weiss@tuwien.ac.at

Tel: +43 1 58801 151611

Fax: +43 1 58801 16199

**
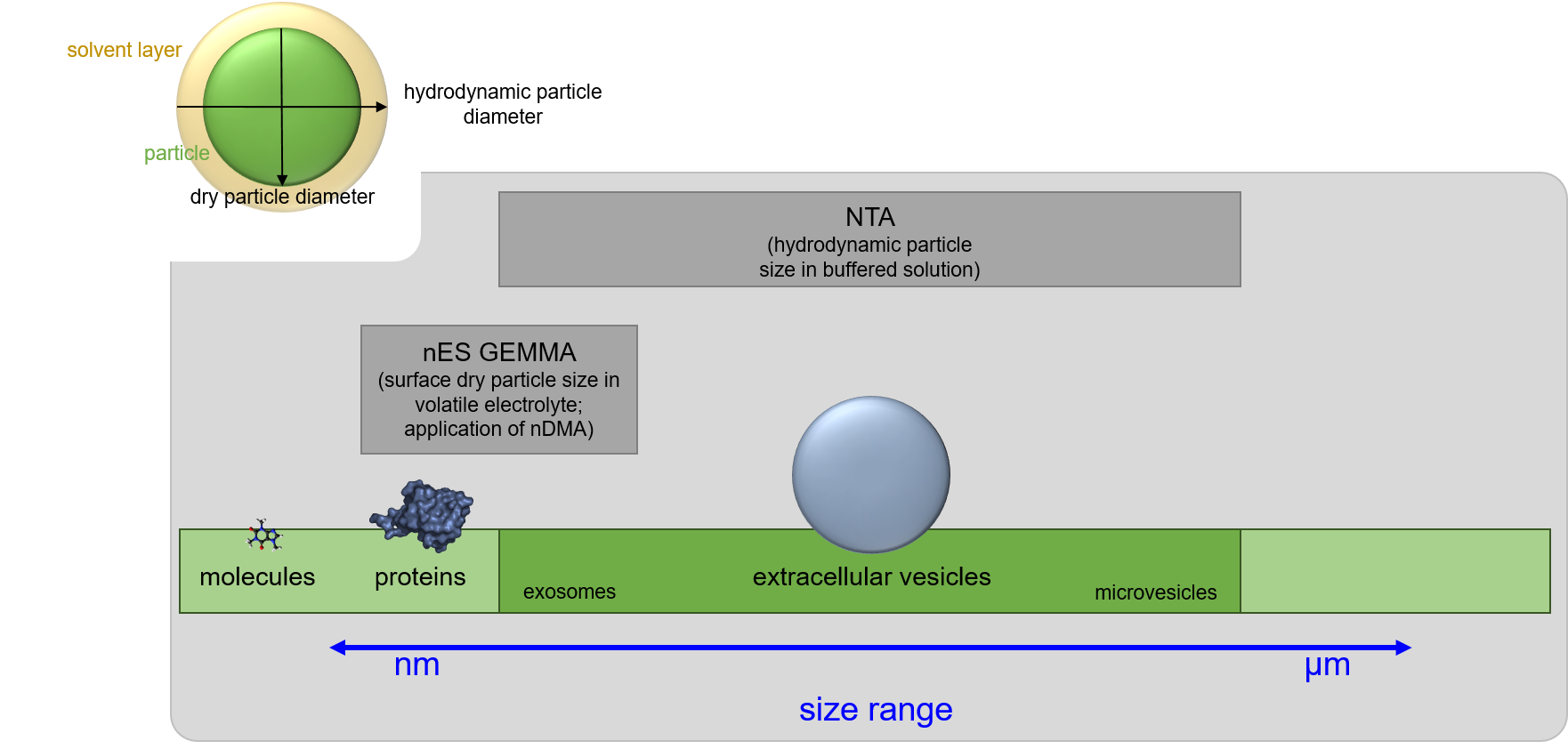
**

**Electronic Supplementary Figure S1: Schematic drawing of size ranges covered by nES GEMMA and NTA techniques.** For nES GEMMA, a nDMA setup is regarded.

**
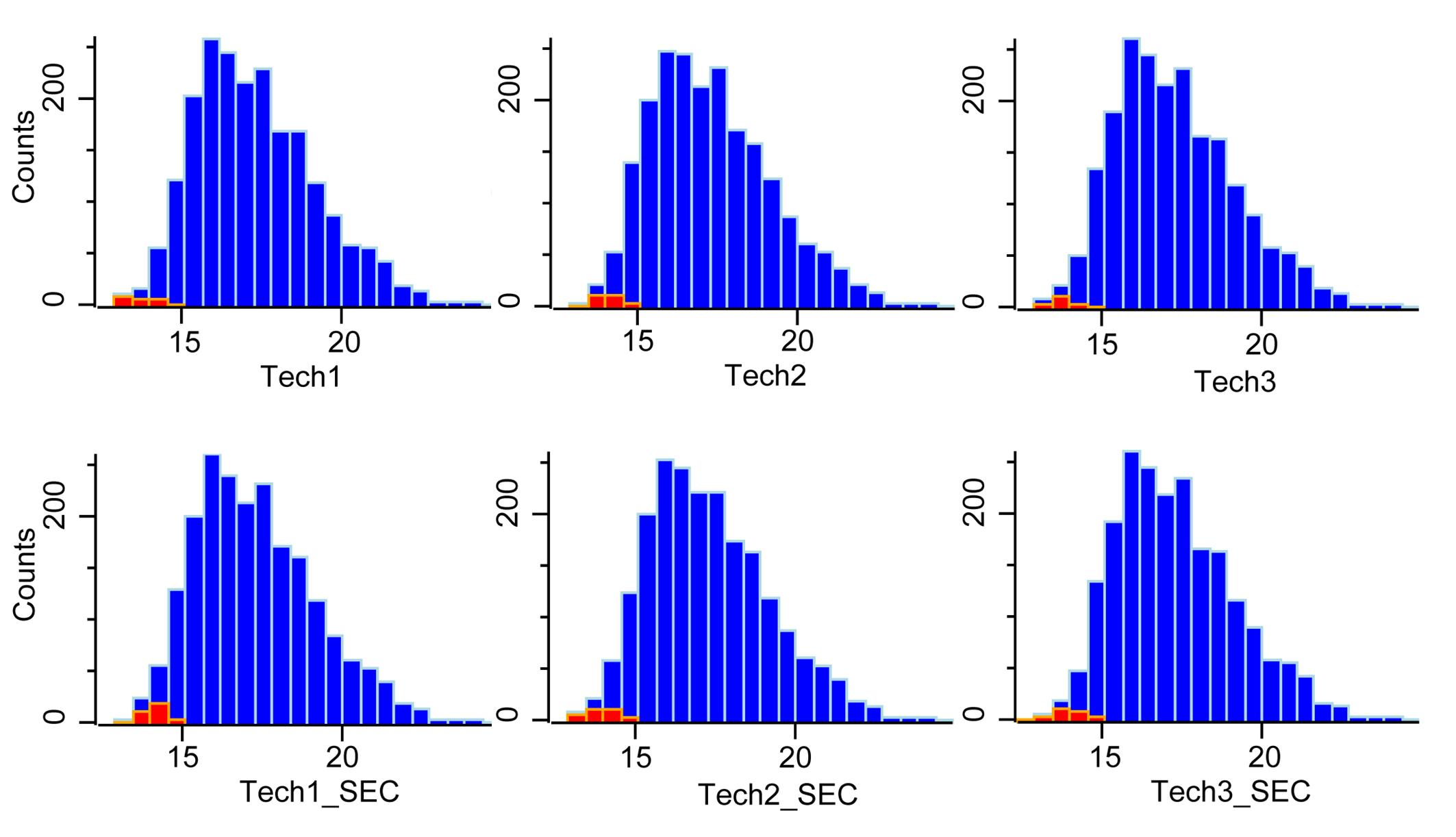
**

**Electronic Supplementary Figure S2: Histograms of intensities of quantified proteins from LC-ESI-MS.** Measured proteins are depicted in blue while imputed proteins are depicted in red - a normal distribution is suggested.


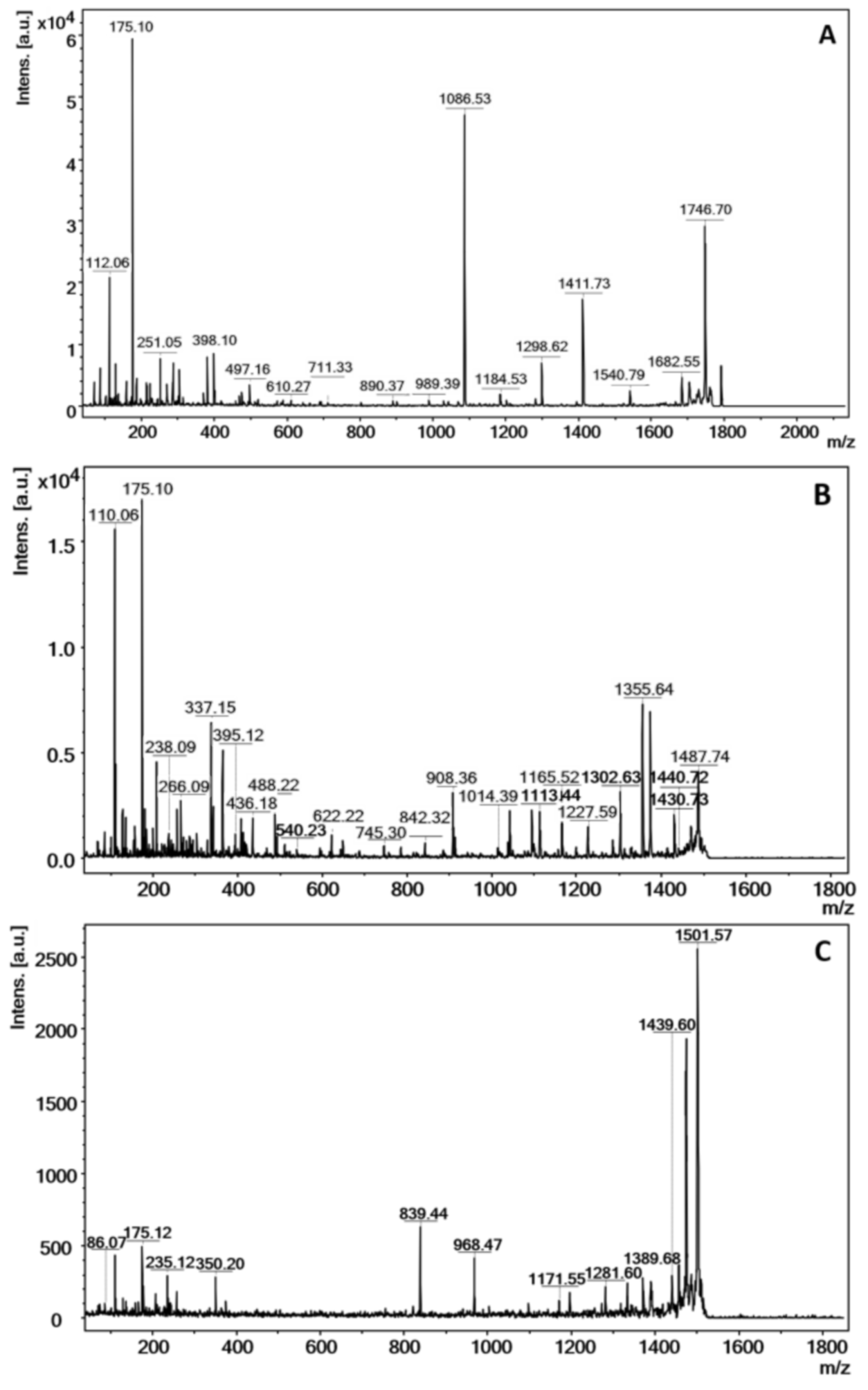


**Electronic Supplementary Figure S3: MS/MS spectra of identified protein components of EV isolates**. The three identified proteins are: (A) β-actin (like) protein, (B) haemoglobin and (C) α-2-macroglobulin. (A) and (B) occur in larger EVs (EV20k), while (C) is found in smaller EVs (EV100k).

**Supplementary Table 1:** The complete list of proteins identified via LC-ESI-MS can be found as an individual File.
